# Ethics, climate change and health – a landscape review

**DOI:** 10.12688/wellcomeopenres.19490.1

**Published:** 2023-08-14

**Authors:** Julian Sheather, Katherine Littler, Jerome A Singh, Katharine Wright

**Affiliations:** 1 Medecins Sans Frontieres Operational Centre Amsterdam, Amsterdam, North Holland, The Netherlands; 2 World Health Organization, Geneva, Geneva, Switzerland; 3 Howard College School of Law, University of KwaZulu-Natal, Durban, KwaZulu-Natal, South Africa; 4 Dalla Lana School of Public Health, University of Toronto, Toronto, Ontario, Canada; 5 Academy of Science of South Africa, Lynnwood Ridge, Gauteng, South Africa

**Keywords:** Ethics, climate change, health, public health

## Abstract

Anthropogenic climate change is unequivocal, and many of its physical health impacts have been identified, although further research is required into the mental health and wellbeing effects of climate change. There is a lack of understanding of the importance of ethics in policy-responses to health and climate change which is also linked to the lack of specific action-guiding ethical resources for researchers and practitioners. There is a marked paucity of ethically-informed health input into economic policy-responses to climate change—an area of important future work. The interaction between health, climate change and ethics is technically and theoretically complex and work in this area is fragmentary, unfocussed, and underdeveloped. Research and reflection on climate and health is fragmented and plagued by disciplinary silos and exponentially increasing literature means that the field cannot be synthesised using conventional methods. Reviewing the literature in these fields is therefore methodologically challenging. Although many of the normative challenges in responding to climate change have been identified, available theoretical approaches are insufficiently robust, and this may be linked to the lack of action-guiding support for practitioners. There is a lack of ethical reflection on research into climate change responses. Low-HDI (Human Development Index) countries are under-represented in research and publication both in the health-impacts of climate change, and normative reflection on health and climate change policy. There is a noticeable lack of ethical commentary on a range of key topics in the environmental health literature including population, pollution, transport, energy, food, and water use. Serious work is required to synthesise the principles governing policy responses to health and climate change, particularly in relation to value conflicts between the human and non-human world and the challenges presented by questions of intergenerational justice.

## Introduction

According to the World Health Organization, climate change is the single biggest health threat facing humanity. ^1^ The health impacts, both current and projected, of climate change are increasingly well understood. Health impacts are also increasingly acknowledged as central to any coherent policy response to climate change. ^2^ The drivers of climate change lie in human economic activity broadly conceived, particularly, but not exclusively, the use of fossil fuels (methane from food production is also a significant contributor). Successful policy responses to climate change therefore need to address health impacts in a huge range of policy areas related to human activity that directly or indirectly generate greenhouse gases (GHGs). Although climate science and economistic disciplines have predominated in policy discussion of, and response to, climate change, given the centrality of human health and wellbeing, it is critical that policy making is properly informed by health expertise. ^3, 4^ This involves health engagement in fields including, but by no means limited to, the global economy and governance, energy policy, food and nutrition, pest control, pollution, the built environment, transport policy, land usage, water usage and management, population and the use of technological responses to climate change.

Although each of these areas of concern requires scientific and technical expertise, policy decision-making in this area is irreducibly ethical. ^5^ Reflection on anthropogenic climate change has forced us to ask philosophically demanding questions about topics as diverse as whether the earth’s ecosystems—or parts thereof—have intrinsic as well as instrumental value; what present-day polluters may owe to future generations, and whether countries most at risk of the harms of climate change are owed reparations by historical polluters. Such questions are straightforwardly ethical. On a slightly more granular level, as climate adaptation and mitigation policy and practice develop, and their benefits and burdens are identified and allocated, such trade-offs will give rise to enduring and complex ethical challenges – particularly, but by no means exclusively to do with justice and fairness. ^6^ From a health perspective, these will include questions about what part the current health burdens of climate change should play in decisions regarding reparation. Health, that is, has a critical part to play in urgent debates about Loss and Damage resulting from climate change. It is essential therefore that any health-related inputs into climate change policy are sensitive to relevant ethical considerations. ^7^ As Donald Brown has written ^8^, attempts to gain general ethical traction in environmental policy-making have been less than successful, and what applies to broader environmental ethics inevitably holds true for health-related ethics in climate change.

The question inevitably arises as to what kinds of ethical considerations, principles or approaches should govern health-related interventions into such wide-ranging and interdisciplinary climate policy. Ethical principles or approaches governing *clinical* ethics, although well established, have a proper focus on respect for the dignity and autonomy of individual patients. But this may not get us very far. Although they draw attention to issues of important moral concern, many of the major ethical challenges in climate change have to do with the allocation of benefits and harms across populations, countries, regions and even generations, and these issues are not front and centre in clinical bioethics. Public health ethics, with its focus on the health of populations certainly looks more promising. Questions of justice are fundamental to public health ethics, as well as the need to adjudicate between individual rights and liberties and the interests of groups and populations. Public health practitioners are also experienced at working with many of the health impacts of the policy areas listed earlier, such as pollution, transport and the built environment.

This review sketches out the current landscape regarding ethics, climate change and human health, including interventions—and research into interventions—aimed at both mitigating and adapting to its health-related impacts. Reviewing this area is challenging. The interaction between health and climate change is both scientifically and theoretically complex. Policy engagement is necessarily inter-disciplinary, requiring input from both climate and health sciences. The scale and complexity of the ethical challenges are such that leading thinkers have for some time argued that it requires a paradigm shift in ethical thinking. ^9^ Furthermore, it is simply not feasible to undertake a single comprehensive review of the combined fields of climate change, the impact of climate change on health, and associated ethical issues. As one recent systematic review of the literature focusing solely on the impact of climate change on human health stated:

*Research on climate and health takes place across various disciplines and silos, representing a fragmented landscape of niche discourses that hinders efforts to synthesise key insights and identify trends and evidence gaps. Second, exponentially increasing literature means that conventional evidence synthesis methods that typically require considerable human resources to manually collate and screen literature are no longer sufficient or feasible.* ^10^

Acknowledging these serious challenges, this landscape review is divided into several parts. The first discusses—briefly—the most up to date scientific literature regarding climate change as set out by the IPCC and includes a discussion of scientific uncertainty concerning climate stability. The second engages with identified and anticipated health impacts of climate change, relying on recent syntheses of the literature. The third section, which was the focus of the supporting literature search, ^11^ discusses the ethics of health and climate change with a focus on policy and implications for researchers and public health practitioners broadly understood. The final section discusses available ethical frameworks that can help inform practice at the interface of climate change and health. Given the length and disciplinary breadth of this area, it takes a modular approach. Each of the separate sections are designed to be able to stand alone.

This paper forms part of a larger WHO project designed to embed ethics in climate change health policy and practice, including all aspects of associated research. Without ethical awareness, interventions in this area will struggle – failing to identify critical competing interests, value conflicts and unintended consequences. Policy and practice, including research practice, will be seen as unfair or otherwise unethical. The project will deliver a range of supporting tools and materials clarifying the ethical considerations for those involved in policy, research or practice in health and climate change. They will help address health-related ethical issues across the mitigation, adaptation and ‘Loss and Damage’ policy arenas. Research here will include the ethical setting of research priorities, along with research conduct more broadly.

## Part one: climate science – what do we know about climate change?

In 1988, the World Meteorological Society and the United Nations Environment Program established the IPCC (Intergovernmental Panel on Climate Change) ^12^. Its purpose is to provide governments ‘at all levels with scientific information that they can use to develop climate policies.’ ^13^ Its membership includes scientists from 196 countries who peer review emerging research to provide the most authoritative, independent and up to date scientific data on human-induced climate change. The IPCC is currently in its sixth reporting cycle (AR6). Technical and scientific advances since the fifth reporting cycle (AR5) include ‘improvements in observationally based estimates and information from paleoclimate archives… new climate model simulations, new analyses, and methods combining multiple lines of evidence’ which have led to ‘improved understanding of human influence on a wider range of climate variables, including weather and climate extremes.’ The IPCC formulates its findings either as statements of fact or provides an assessed level of confidence. Key points from the Sixth Assessment Report (AR6) ^14^ in relation to the physical science base of climate change are given below. ^15^

According to AR6 it is *unequivocal* that human activity lies behind the warming of our oceans, land and atmosphere. AR6 estimates the *likely* range of anthropogenic surface temperature increase from 1850–1900 to 2010–2019 is 0.8°C to 1.3°C, with a ‘best estimate’ of 1.07°C. Human activity is *very likely* the primary cause of these increases since 1971. Land biosphere shifts since 1970 are in line with global warming: climate zones have shifted toward the poles in both hemispheres.

According to AR6, recent climatic changes —‘are unprecedented over many centuries to many thousands of years.’ and ‘global surface temperature has increased faster since 1970 than in any other 50-year period over at least the last 2000 years (high confidence). Almost all the world’s glaciers are simultaneously retreating, an event unseen for at least 2000 years. Global sea levels have risen faster since 1900 than during any century in the last 3000 years, and our oceans have warmed faster than at any time since the end of the last ice age.

Anthropogenic climate change is driving extreme weather across the globe, including droughts, tropical storms, heatwaves and extreme rainfall. AR6 now regards it as ‘ *virtually certain’* that heatwaves are more frequent and extreme since the 1950s, while extreme cold is much less frequent. 

On the basis of all envisaged scenarios for the emission of GHGs, temperatures will continue to increase until at least 2050. Without significant reduction in GHG emissions, the coming decades will see global warming in excess of 2°C. Semi-arid regions, along with parts of South America, will likely see the highest increases in the temperatures of the hottest days – as much as twice the global warming average. There remains the possibility of ‘low-likelihood’ or ‘black-swan’ events, such as sudden changes in ocean circulation or the collapse of the Greenland ice sheet with potentially extreme global consequences.

### Progress toward climate change mitigation

Progress toward prevention of hazardous anthropogenic climate change through reduction in the emission of carbon and other greenhouses gases has fallen far short of targets set 30 years ago by the UN Framework Convention on Climate Change (UNFCCC). Energy production is still overwhelmingly dependent on fossil fuels—renewables accounting for just over 8% of global energy—the carbon intensity of the world’s energy system has decreased by less than one per cent and energy demand has risen by 59% since the UNFCCC was established. 2021 saw a historical high in energy-related GHG emissions, with current emissions set to increase global temperatures by a catastrophic 2.7% by the end of the century. Even existing country commitments are likely to see global emissions rise to 13.7% above 2010 levels by 2030 – a long way short of the Paris Agreement goals. ^16^ A November 22 briefing in the *Economist* magazine, likely to be highly controversial, argues that the 1.5°C Paris target must now be abandoned as unfeasible. It states:

*Global average temperatures are currently 1.0-1.3°C above the pre-industrial. According to Britain’s Met Office and the World Meteorological Organisation, there is a 48% chance that global average temperatures will be 1.5°C higher than pre-industrial in at least one of the next five years*. ^17^

The combination of legacy atmospheric carbon, the requirement for cheap, economically transformative electricity in poor countries, some of which will need to come from fossil fuels, along with continued backsliding among the biggest producers of GHGs make it inevitable, in the *Economist*’s view, that the 1.5°C target will be missed.

### Adaptation responses

According to the IPCC, climate change adaptation, both at planning and implementation stage are taking place across all sectors and in all parts of the world, generating multiple benefits including in relation to human health ^18^. Adaptation is however unevenly distributed, with noticeable gaps in resource-poor settings. Much adaptation prioritizes immediate and near-term climate risk reduction, at the possible expense of long-term transformational adaptation. The effectiveness of adaptation will however decrease if warming continues to increase.

‘Soft’ limits to some forms of adaptation have almost certainly been reached, but can be addressed by tackling a variety of financial, governance, institutional and policy constraints. Hard limits to adaptation have however almost certainly been reached in some ecosystems. With increasing global warming, losses and damages will increase and it is very likely that more human and natural systems will reach their adaptation limits. ^19^

The IPCC also highlights the risk of current ‘maladaptive’ responses to climate change. These are responses that ‘may lead to increased risk of adverse climate-related outcomes, including via increased greenhouse gas emissions, increased or shifted vulnerability to climate change, more inequitable outcomes, or diminished welfare, now or in the future. Most often, maladaptation is an unintended consequence.’ ^20^ Examples include the use of sea walls or other hard defences against rising sea levels. These ‘reduce space for natural processes and represent a severe form of maladaptation for the ecosystems they degrade, replace or fragment, thereby reducing their resilience to climate change and the ability to provide ecosystem services for adaptation. Considering biodiversity and autonomous adaptation in long-term planning processes reduces the risk of maladaptation.’ ^21^

### Responding to uncertainty

Reliable monitoring and prediction of anthropogenic climate change is essential to the development of global policy responses. Decisions about whether to prioritise mitigation or adaptation strategies, and what costs are reasonable to incur in their pursuit, require at least some confidence in predictive outcomes. As a 2019 editorial in *Nature Climate Change* puts it:

*Governments need to know what will happen to decide how best to invest money and infrastructure; and they need to know what effect policies meant to reduce emissions and slow climate change will have. As more and more countries pass national and international legislation aimed to mitigate climate change, measuring the efficacy of these policies will become a priority that is likely to play a role in their strength and success.* ^22^

A criticism often levelled at climate projections is the persistence of scientific uncertainty – and such uncertainty is often seized upon by climate sceptics, and wholesale climate change deniers, to sow doubt as to the reality of climate change itself. Given the complexity of the earth’s climate it is likely that some degree of uncertainty is inescapable. As decision-making in the face of imperfect information gives rise to ethical challenges, this section briefly addresses scientific uncertainty.

Somewhere in the region of 97% of climate scientists agree with the propositions that climate change is real and that it is caused by anthropogenic emissions of greenhouse gases (GHGs). ^23^ This paper fully endorses that consensus. It remains the case however that although the physical mechanisms behind climate change, and its broad-brush effects, are scientifically well understood, uncertainty remains about the precise scale and timing of many features of climate change. Because the earth’s climate systems are exceptionally complex and interrelated, they are both challenging to predict in detail and vulnerable to large-scale discontinuities, more often called ‘tipping points’ or ‘threshold events.’ ^24^ Such events include the possible collapse of the Greenland ice sheet and the serious disruption of a critical feature of the global ocean circulation system, the Atlantic Meridional Overturning Circulation (AMOC). That the earth’s climate is subject to natural variation over long time spans has also been used to criticise the modelling used by the IPCC and to introduce some scepticism regarding its global warming forecasts. ^25^ It is possible therefore that the impacts of anthropogenic climate change may be less severe than forecast, but they may also be very much more severe. It is for this reason that the IPCC uses an approach based on levels of confidence in its predictions. ^26^ As climate science develops, and as more longitudinal climate data are accrued, the IPCC’s confidence in many of its predictions increases.

As Stephen Gardiner points out ^27^, the IPCC—and many of those who might be regarded as sceptical of at least some of the IPCC’s forecasts—do not regard the data regarding global warming, or that it is the result of human economic activity, to be *uncertain*. As discussed above, the IPCC regards both propositions to be unequivocal. Technically therefore, many of the IPCC’s predictions are not a matter of uncertainty but of *risk*, where risk refers to a situation under which the decision outcomes and probabilities of occurrences are known to the decision maker, with uncertainty referring to a situation where such information is not available. ^28^ As we have seen, the IPCC assigns high levels of likelihood to a range of forecast outcomes.

But even if real uncertainty regarding climate change existed, it is not clear that inaction would be a reasonable response. As Gardiner again points out ^29^, the physical processes that give rise to global surface warming are well understood. Without a naturally occurring greenhouse effect, the earth’s surface would be considerably cooler. The scientific record concerning increased anthropogenic concentrations of GHGs is also clear, and the kinds of surface warming we are seeing in recent decades are precisely what we would expect from knowledge of the greenhouse effect. Although the earth’s climate is subject to natural variation, and its complexity makes unequivocal predictions of specific changes a matter of probabilities rather than certainties, nothing in this suggests that inaction is a viable proposition. The question is not whether we should respond to what we know, but how.

## Part two: the health impacts of climate change – IPCC and *Lancet* Countdown

The literature on the health impacts of climate change is both considerable and increasing at pace. According to a recent systematic review in *Lancet Planet Health*, the literature is now so extensive, ‘it is no longer feasible to collate and synthesise using traditional systematic evidence mapping approaches.’ ^30^ A machine-learning search of Web of Science Core Collections, Scopus and PubMed between 2013 and 2019 on climate and health by the same authors identified—predicted is their word—15,963 studies. In this section we therefore look briefly at IPCC AR6 and *Lancet Countdown* along with recent systematic reviews of the literature on health and climate change.

The health and wellbeing chapter of the IPCC’s Sixth Assessment Report, corroborated by several recent papers, ^31^ presents a sobering and comprehensive account of the health impacts of climate change, which it regards as ‘largely negative at all scales.’ ^32^

Climate-related illnesses, including communicable and non-communicable diseases (NCDs), early deaths, malnutrition, and threats to mental health and general well-being are all rising. Several NCDs are sensitive to climate change as they are exacerbated by dust, small particulates, heat, fire smoke and allergens. Climate-driven involuntary migration and displacement are increasing and are exacerbating violent conflict. The overall impacts are negative with few examples of positive effects. Growing urbanisation is exacerbating the impact of extreme heat, with ageing populations becoming increasingly vulnerable.

Climate change drives food insecurity with risks of various kinds of malnutrition – including obesity-linked malnutrition, and increased vulnerability to disease, particularly in low- and middle-income countries. Extreme climate events straightforwardly threaten wellbeing through their destructive power but also have indirect effects through income loss and displacement of populations. Heat-related morbidity is expected to rise significantly and climate change is anticipated to expand the range of a number of vector-borne diseases.

To these slightly high-level accounts, *Lancet* Countdown 2022 ^33^ offers some more granular health detail. It states that rapidly increasing temperatures have exposed vulnerable populations – adults over 65 and children under one – to 3.7 billion more heatwave days in 2021 than annually from 1986-2005. Heat-related deaths have increased by 68% between 2000-04 and 2017-21, a figure exacerbated by the Covid-19 pandemic. In relation to infectious diseases, coastal waters are becoming more suitable for the transmission of Vibrio pathogens and the number of months suitable for malaria transmission in high altitude areas in the Americas increased by 31.3% and by 13.8% in Africa from 1951-60 to 2012-21 and dengue transmission increased by 12% in the same period. Climate change, particularly in resource-poor settings is undermining many key socio-economic determinants of health – extreme heat led to 470 billion potential labour hours lost in 2021 – equivalent to 0.72% of global economic output, and 5.6% of GDP in low HDI countries. Food security is being undermined by climate change, acting alongside other crises. Higher temperatures reduce crop yields – the maize growing season was an average of nine days shorter in 2020. Spring and winter wheat growth seasons were six days shorter. Research suggests that an additional 98 million people experienced moderate to severe food insecurity arising from extreme heat in 2020 than in the years 1981–2010.

Despite what can seem to be overwhelming bad news, Lancet Countdown nevertheless highlights the significant benefits a health-related response to climate change can deliver:

*In this pivotal moment, a health-centred response to the current crises would still provide the opportunity for a low-carbon, resilient future, which not only avoids the health harms of accelerated climate change, but also delivers improved health and wellbeing through the associated co-benefits of climate action. Such response would see countries promptly shifting away from fossil fuels, reducing their dependence on fragile international oil and gas markets, and accelerating a just transition to clean energy sources. A health-centred response would reduce the likelihood of the most catastrophic climate change impacts, while improving energy security, creating an opportunity for economic recovery, and offering immediate health benefits. Improvements in air quality would help to prevent the 1·2 million deaths resulting from exposure to fossil fuel-derived PM (particulates) and a health-centred energy transition would enhance low-carbon travel and increase urban green spaces, promoting physical activity, and improving physical and mental health. In the food sector, an accelerated transition to balanced and more plant-based diets would not only help reduce the 55% of agricultural sector emissions coming from red meat and milk production … but also prevent up to 11·5 million diet-related deaths annually … and substantially reduce the risk of zoonotic diseases.* ^34^

### Health and climate change – systematic reviews of the literature

Two important recent papers – Berrang-Ford *et al*.’s 2021 systematic mapping of global research on climate and health using machine learning ^35^, and Rocque *et al.*’s 2021 overview of systematic reviews on the impact of climate change on health – provide a fascinating overview of recent research. According to Berrang-Ford, of the 15,963 papers identified, the overwhelming majority (84%) focus? on health impact studies, with only a small number looking at human responses to climate change – 10% looking at mitigation and 7% at adaptation, with ‘few studies focused substantively on the benefits to climate change mitigation or adaptation in the health sector.’ ^36^ The regional distribution of studies was interesting, with 79% of studies that incorporated a location being focussed on high-income and upper-middle income countries, particularly China. The number of studies focussed on high-income countries was 10-times those focussed on low-income countries. ^37^ Berrang-Ford *et al.* used a topic-modelling approach based on five categories: Key hazards, health impacts, mediating pathways and risk modifiers, options and responses and ‘other’.

Primary hazard topics were extreme events—including floods (6% of 15,963 articles), hurricanes (5%), heatwaves (5%), drought (4%), dust storms (3%) and wildfires (4%)—and air quality, including particulate matter (12%) and nitrous oxide and vehicle and ozone emissions (10%). Meteorological variation as a source of health hazard included changes to rainfall patterns (12%), extreme or increasing temperatures (9%) and shifting seasonality (15%). Perhaps unsurprisingly, a range of regional variations was identified in relation to hazards. The impact of particulates on health quality was identified as important in Asia and Europe. Likewise, extreme events, particularly hurricanes, were recognised as important in North America, heatwaves were a major topic in Europe and Oceania, with Africa and Latin America particularly concerned with rainfall and meteorological variation. ^38^

The major health topics identified by Berrang-Ford, include heat stress, air quality, infectious diseases and all-cause mortality. There was a strong focus on respiratory impacts – including air pollution (16% of 15,963 articles), respiratory viruses (6%), asthma (3%), pollen and allergies (2%) – heat stress (9%), vector-borne infectious diseases such as dengue (5%) and malaria (5%), along with influenza (2%), cholera (1%) and leptospirosis (1%). Public health was a major focus (19% of 15,963 articles), along with concerns about the increasing impact of climate change on health facilities. Other health foci included water and sanitation (7%), maternal and child health (7%), food insecurity and agriculture (5%), mental health (5%) and occupation health and injury (5%).

Among the principle mediating pathways identified by Berrang-Ford were social vulnerability, heat risk and infectious disease exposure in urban pathways. Age and sex were the principle risk modifiers (15% of 15,963 articles) and were in the top three topics for all regions. Health in urban areas was identified as an emergent topic (11%), particularly risks arising from urban heat islands in Europe and urban exposure to infectious diseases in Latin America. Socially mediated vulnerability (9%) was an important topic in all regions, particularly North America and Oceania, with the exception of Asia. China was the only geographically based topic, with a considerable focus on respiratory health and air quality. Building design (4%) and rural households (7%) were important mediating pathways, with a focus on air quality and internal stove usage, with rural households being the most important pathway in Africa. ^39^

In relation to the literature on mitigation and adaptation, energy policy and GHG emission pathways were the major topic, appearing in the top three for all regions apart from Latin America and Oceania. Discussion of mitigation also included modelling for future climate scenarios and routes to emissions reduction. The adaptation literature focused on reducing disaster risk (7% of 15,963 articles), community resilience (10%) and adaptation policy and practice (9%).

Surveying their results, Berrang-Ford *et al.* were struck by the ‘poor integration of research on impacts, mitigation, and adaptation across key topics.’ Using a visual topic map, where similar topics are pictured closer together and dissimilar topics further apart, the authors found:

*The topic map shows a large number of clusters of impact-related topics, including several focused on specific health outcomes (eg, malaria, influenza, suicide, and stroke) that are highly clustered and separate from other topics. CCVW (Climate Change, Climate Variability and Weather)-related topics, such as seasonality, meteorology, and temperature, show less distinctive clustering. Of the health topics, heat stress and air quality appear to be the most strongly integrated with CCVW-related topics. Mitigation topics are fewer and clustered together, with substantial overlap between mitigation and air pollution topics. Adaptation clusters are relatively uncommon and under-represented. Notably, there was no substantive overlap of adaptation topics with mitigation topics, indicating negligible attention to co-benefits and co-risks across these two dominant response options. The health areas most strongly clustered with adaptation topics include food and mental health, with many health topics showing negligible proximity to adaptation clusters*. ^40^

Berrang-Ford *et al.* found that in high income areas, the literature focussed on the impacts of climate change on hospital admissions, chronic disease and health service pressures, including the impact of heat, air quality and extreme events. Research in lower income settings was heavily concerned with infectious diseases, with a strong secondary focus on food, nutrition and child and maternal health.

Possible options and response to the health impacts of climate change in the literature focussed on co-benefits between respiratory health improvements and mitigation, along with potential co-benefits between mitigation, chronic and infectious diseases and all-cause mortality. There were co-benefits between energy policy, air pollution and short-term health impacts, but stronger longer-term health benefits were identified as co-occurring with changes in greenhouse gas pathways, particularly in relation to heat stress and infectious disease. Mental health and adaptation co-occurred, particularly in relation to managing disaster risk and strengthening community resilience, as did maternal and child health. Adaptation policy co-occurred with health system demand, along with water, sanitation and hygiene and food and nutritional health. ^41^

Among the gaps in the literature identified by Berrang-Ford *et al.* include:

- The under-representation of literature from central Asia, north and central Africa and South America
- Disconnected foci of research—in Africa for example, research is largely focussed on vector-borne disease and public health systems, despite the need for research into the impact of climate change on maternal and child health, respiratory infections and nutrition—the first, second and 11 ^th^ causes of DALYs in Africa in 2019
- An insufficient focus on mental health, including on the impacts of agricultural shifts and extreme events and the impact of climate change driven migration on social cohesion
- An underrepresentation of the social determinants of climate-driven health impacts, and on points of intervention in the literature
- The paucity of evidence on the health effects of mitigation and adaptation – which will limit the development of evidence-based pathways to reduce climate change impacts on health.

Rocque *et al.* undertook an overview of systematic reviews of the impact of climate change and health. ^42^ Their headline findings include:

- Meteorological impacts, mostly temperature and humidity, were the most common impacts studied, which suggests that further research is required on the impact of other aspects of climate on health, including direct and indirect impacts of rising temperatures, such as drought and wildfire smoke
- Systematic reviews prioritise physical health outcomes, principally infectious diseases, mortality, and respiratory, cardiovascular and neurological impacts, suggesting that the research focus should be broadened out to include a wider range of health outcomes. These should include the impacts of climate change on mental and broader social well-being.
- Although mental health impacts were largely focussed on the direct effects of extreme weather events, the longer-term indirect mental health impacts such as eco-anxiety and climate depression are becoming more prevalent
- There is a stark geographical separation in the country affiliation of first-authors, with over 75% being affiliated to institutions in Europe, Australia or North America
- In addition to well-established associations between climate change and negative health outcomes, less-frequently studied associations include those between climate change and increased use of health services, some mental health impacts, nutritional deficits and worsening occupational health
- The existence of limited and conflicting evidence—along with absence of evidence—concerning the impact of climate change on health suggest the need for further research. Associations are complex and tracking causal relationships between climate change and health imposes important methodological challenges ^43^
- The health framing of climate change is insufficiently used in climate-related communications, thereby missing opportunities to increase engagement with the climate crisis. There is significant scope for exploring the role of key stakeholders, such as health care professionals and policy makers, with a view to strengthening their roles in climate change adaptation and mitigation and their voice in climate-related communications.

## Part three: health, climate change and ethics

For this section of the landscape review, a search was undertaken for current literature on ethical issues related to human health and environmental and/or climate change, with a particular focus on articles related to policy development. ^44^ The purpose was to gain an understanding of the extent to which some form of action guiding ethical reflection was available for those working in policy and research at the interface of climate change and human health. This would then permit the identification of both gaps in the literature and areas for possible future work.

The thematic spread of the papers was interesting. The 133 papers were manually sifted for primary and secondary thematic foci, using an informal and indicative categorisation scheme drawn from the literature itself. As many of the papers had more than one main focus, the following distribution of papers amounts to more than 133: Adaptation (3 papers), biosecurity (2), co-benefits (4), country or region specific (17), climate change economics (10), energy production and use (1), ethics and human rights (27), effects of heat (1), justice and fairness (18), mitigation (5), one health/planetary health (10), policy, negotiation and communication (13), pollution (2), population (2), public health including the use of public health tools (25), research (5), the role of medicine, health professionals and health services (10), the use of new technologies to tackle climate change (10) and urbanisation (1). A catch-all miscellaneous category had seven papers including topics such as how preparing for climate change can revitalise democracy, the sociology of incorporating human beings into their environment, and the natural environment as an object of public health law. Of the 17 papers with a specific country or regional focus, five were in sub—Saharan Africa, three in South Africa, eight in HDI countries, including Canada, the USA and the Netherlands, and four were in Asia. We look first at the higher-level normative questions identified in the literature before looking in more detail at policy-related reflection.

### High-level normative questions

Analysing in more detail the specifically normative literature searched—which includes references to ethics, human rights and justice/fairness—it is plausible to state that the main high-level areas of concern in relation to ethics and climate change are reasonably well defined and largely agreed upon in the literature – even if, as Gardiner points out, there remains a serious lack of robustness in our best theoretical approaches to these issues. ^45^ The reality of anthropogenic climate change is accepted, as are the increasingly serious burdens for current and future generations. The following areas of ethical concern were addressed in the ethics and human rights literature surveyed.

***Collective action problems***

It is widely accepted that climate change presents an extremely challenging version of a collective action problem. No matter where they originate from, greenhouse gases rapidly mix and disperse, affecting the global climate. This leads to a ‘tragedy of the commons’, where individual nation states, governed by the principle of Westphalian sovereignty, act rationally in their own economic short-term self-interest, thereby irreparably damaging the global environmental commons. (Westphalian sovereignty refers to a norm of international law that prioritises territorially limited national interests over international ones, presenting serious challenges to efforts to address the climate crisis. ^46^)

Such a description does not however capture all that is morally relevant about climate change. As Gardiner points out, it ignores the fact that those least responsible for historical—and to an extent current—emissions are likely to suffer worst. Many of them are in regions likely to be highly impacted by climate change and are economically less able to adapt to its effects. Calling climate change simply a tragedy of the commons neglects these fundamental questions of justice and fairness. ^47, 48^

***Intergenerational justice***

To this Gardiner adds a temporal perspective:

*Once emitted, a substantial proportion of climate emissions typically remain in the atmosphere for hundreds of years, and some persist for tens – even hundreds – of thousands. This means the current generation takes benefits now, but spreads the costs of its behaviour far into the future. Worse, many of these benefits are comparatively modest (eg those of bigger and more powerful vehicles), and many of the projected costs are severe, even catastrophic.* ^49^

Another key challenge is therefore the extent to which we are obliged to consider the wellbeing of future people when making decisions that affect our climate. It would be in the interests of future generations and some vulnerable species if GHG emissions were substantially reduced as quickly as possible to minimize future climate impacts. However, this may be costly for the current generation while having few tangible benefits for them. Secondly, given the serious harms arising from climate change, intergenerational justice asks us to consider whether past climate injustices give rise to reparative obligations to those in the future who will be harmed by them. ^50^

One familiar challenge to questions of intergenerational justice is what philosophers call the non-identity problem. ^51^ Simply put, this asks how future persons can be harmed, or disadvantaged, by acts or social policies which are necessary conditions of their coming into existence. Unless their lives are unspeakably burdensome such that they are better off not existing, any policy that results in their existence straightforwardly benefits them.

***Who is responsible for what?***

Another central moral challenge is the question of dispersed responsibility. Human activity has been changing the planet and its ecosystems for thousands of years. The industrial revolution, and the large-scale use of fossil fuels that drove it, started in Europe at some point in the mid-18 ^th^ century. Many generations, from many nations, have therefore contributed to atmospheric carbon concentrations. Given that many nations risk being devastated by changes in climate they have only marginally contributed to, is it therefore morally plausible to talk about possible reparations for those harms, and if so, who should be held accountable?

***Individual action and collective problems***

A linked ethical problem arising from the apparently intractable nature of these political challenges, is the perceived futility of individual actions. Where nation-states are aggressively competing to deplete the global commons, what is the point of individual actions that seek to limit climate change? What ethical obligations do individuals have to act where governments fail to do so? And further, how can we develop action-guiding principles with sufficient normative power to guide virtuous individual decision-making in the face of national irresponsibility? ^52^

***The value of the non-human world***

A further major normative challenge has to do with the moral standing of the non-human world, including its non-human biotic and abiotic components. ^53^ Given that our health and wellbeing depend upon healthy ecosystems, they have clear instrumental value: healthy ecosystems are necessary for the fulfilment of human ends, needs, interests or preferences. ^54^ However, several important environmental thinkers seek to locate some form of *intrinsic* value in ‘nature’ and natural ecosystems. Even if human beings no longer existed, the argument runs, the earth’s ecosystems, its landscapes, seas and oceans would still have moral value. ^55, 56^ Critical questions here include what kind of moral value accrues to the non-human world, and how to adjudicate where clearly identifiable human interests conflict with the interests of non-human parts of the biosphere.

***Procedural and distributive justice***

The harms of climate change are unfairly distributed, falling disproportionately on populations already disadvantaged and lacking the economic and social resources to adapt. Questions of justice are therefore central, particularly the just division, fair sharing, and equitable distribution of the benefits and burdens of climate change, along with the benefits and burdens of adaptation and mitigation policies and the allocation of responsibilities to address them. This feeds into one of the most urgent current political debates in climate change: the question of Loss and Damages. This refers to the fact that climate change is already incurring significant economic and non-economic costs—including in relation to health—for many, if not all nations. As these costs are unfairly distributed, falling with particular severity on countries and regions economically less capable of responding, Loss and Damages also refers to efforts to ‘avert, minimise and address loss and damage associated with climate change impacts, especially in developing countries that are particularly vulnerable to the adverse effects of climate change.’ ^57^

For our purposes justice can be roughly grouped into two categories: procedural justice—which has to do with the fairness of a given decision-making process including fair, transparent and inclusive decision-making—and distributive justice, which is concerned with how goods, services, and entitlements should be fairly apportioned. 

Aspects of justice relevant to climate change include:

- Inequalities in who has *contributed* to climate change;
- Inequalities in who is most *harmed* by climate change; and
- Opportunities to rectify injustice while addressing climate change. ^58^

Questions of how to address questions of global distributive justice in relation to climate change remain amongst the most practically and theoretically stubborn.

***The poverty of theory***

A final point, as touched on above, is that although there is some consensus on the nature of the major ethical questions presented by climate change, we lack coherent and robust ethical theories to address these problems. Gardiner again states:

*Climate change brings together many areas in which our best theories are far from robust, such as intergenerational ethics, global justice, scientific uncertainty, and humanity’s relationship to nature. The problem here is not that we do not have any guidance at all. For example, the idea that imposing catastrophe on the future for the sake of our own modest benefits is not a defensible way to behave is a relatively secure basic ethical intuition. Rather, the problem is that it is difficult to move beyond those basic intuitions to deal with the details and we are too easily distracted by counterarguments, especially from theories that have merits in other contexts, but fail to take the future seriously enough.*

### Ethics in health and climate change policy

The need to ensure ethically-informed input from health into all areas of climate policy has been established earlier in the paper. This is supported in the literature by an important early paper by Singh. ^59^ (A health perspective has also been identified as particularly effective in driving advocacy and public awareness of, and interest in, the climate crisis. ^60^) As we have seen, the health impacts of climate change in general, and the health impacts of specific aspects of climate change, are well represented in the public health-related literature, with the exception of mental health and wellbeing impacts. ^61, 62^ This reinforces the findings discussed in section two above. ^63^

Although the extant literature reveals an awareness of significant normative challenges in the health-related policy—and research—response to climate change, it also suggests that research and policy in this area struggles with the limits of available ethical theory. This may be why methods for practically implementing these normative concerns are less well developed. Although, for example, questions of justice and fairness were central themes in 10 of the 133 papers we identified—and were important dimensions to the 27 papers directly dealing with ethics and human rights—pathways and policy options for the practical realisation of these concerns, particularly for public health practitioners and researchers wrestling with practical aspects of health-related climate policy, were less evident.

Donald Brown, in an important essay, directly addresses the failure of environmental ethics to achieve traction in environmental policy making. He draws attention to the reliance of public policy making on instrumental rationality, and the failure of academic ethicists to confront the requirements of practical policy-making or to engage effectively with advocates, policy-makers and the media. He also refers to the predominance of scientists and economists in government policy-making and their lack of training in ethics. ^64^

Exceptions to the lack of a clear policy focus include an interesting 2018 paper by Filho *et al.* ^65^ which examines the health impacts of extreme climate events on a multi-regional spread of countries—Bolivia, Uruguay, Cameroon, Ethiopia, Tanzania, Austria, Malaysia and Australia—and outlines some practical, policy-oriented approaches to mitigation and adaptation with a particular focus on justice-related responses in resource-poor settings. Despite the focus on justice, the importance of identifying and responding to broader ethical aspects of climate change policy was not developed in the paper.

Particularly striking in the literature is the lack of sustained ethical reflection on policy relating to major themes in environmental health ethics – areas in which an informed public health response is critical. We identified only single papers in energy production and usage ^66^, the health effects of heat ^67^ and health-related climate change research in urban areas. ^68^ We identified only two papers relating to pollution ^69^ and two relating to population ^70^, both critical areas of climate-related public health concern. The health-related ethics of technological responses to climate change—10 papers—was also surprisingly under-represented. There were four papers related to genetic engineering, four on climate engineering and two focussing on the use of nanoparticles. It is plausible that a different configuration of search terms might have elicited further papers, and more work is no doubt required here, but the findings are still striking.

The ethics of public health practice in relation to climate change was well-represented in our findings – 25 out of 133 papers. Key themes, as anticipated, include the modelling of climate change health impacts and the development of health indicators ^71^. There was some promising, if underdeveloped recognition of the importance of health and public health policy input into less-conventional areas in a small number of papers, including the necessity for public health input into trade policy ^72^, and the importance of introducing a public health perspective into environmental impact assessments. ^73^ The literature nonetheless suggests that when it comes to key areas of environmental health ethics, action guiding material—rather than just a limited description of specific issues of ethical concern—is underdeveloped.

The role of health professionals in both identifying the links between climate change and human health, and communicating the health-related impacts of climate change to both patients and policy makers was evident. ^74^ International co-operation between health professionals was identified as vital to addressing global equity in climate change related health policy. ^75^ This is an area that warrants further exploration, including in relation to the need to de-carbonise health services globally.

We identified 10 papers with a primary focus on one-health approaches to climate change. These are important normatively and practically, as they open up questions about the moral value of the non-human world and point to fertile areas for achieving co-benefits between climate change mitigation and human health and wellbeing – essential if the global public health gains of the last century are not to be overturned. As Almada *et al.* state:

*Collaboration* (is required) *across a broad swath of scientific disciplines as well as with policy makers, natural resource managers, members of faith communities, and movement builders around the world. Only through forging this collaboration can we build a rigorous evidence base of scientific understanding as the foundation for more robust policy and resource management decisions that incorporate both important environmental and human health outcomes.* ^76^

One paper—a PhD thesis—usefully pointed to challenging ethical questions arising in a one-health approach, most obviously the potential for ethical conflict between the value of human and non-human parts of the biosphere. ^77^

We have touched upon the preponderance of scientific and economic disciplines—to the detriment of ethics—in climate change policy. A range of topics emerged in the 10 papers identified with a primary focus on economics. Interestingly, Scovronick *et al.* make a strong claim for the irreducibly ethical nature of economic assessments of health benefits, and the challenges presented by global and intergenerational justice to valuing health impacts of climate change, but such a focussed ethical concern was an outlier. ^78^ Other topics included the use of fiscal measures to promote public health ^79^, the use of cash transfers to promote public health in the context of climate change ^80^ and, as discussed earlier, the importance of public health professionals engaging with trade negotiations. ^81^ There is a striking paucity of ethically informed health input into economic and scientific policy-responses to climate change – an area of important future work.

There were surprisingly few papers with a primary focus on ethics and research in climate change and health, with only five identified. Two were focussed on climate change-related research in specific locations – one on research into climate, heat and health in South Africa ^82^, the other looking at climate change research priorities in Georgia, USA. ^83^ Barbosa *et al*. set out an interesting scoping review protocol to be used to identify whether research into the impact of climate change on health in urban areas was suitably inter-disciplinary and ‘holistic’. ^84^ An interesting 2012 scoping review to determine how well research into climate change and human health met the needs of policy makers found that quantitative studies were rare, and there was a deficit of adaptation studies and studies into most resource-poor regions. ^85^

Overall, our survey of the interface between climate change, human health and ethics found a picture of a fragmented, sporadic and underdeveloped field of study. Key gaps include:

- insufficient general understanding of the importance of ethics in policy-responses to climate change
- a lack of specific action-guiding ethical reflection for health practitioners active in climate change policy
- an under-representation of mental health and general wellbeing in the health impacts of climate change
- a lack of literature from low HDI countries and an over-representation of high HDI countries
- a paucity of ethical commentary and reflection on a range of key topics in environmental health literature, including population, pollution, transport, energy, food and water use
- a shortage of discussion of inter-disciplinary ethics in climate change
- a lack of theoretical coherence across the domains of ethics.

## Part four: ethical frameworks in health and climate change

An established approach to the navigation of ethically complex and contested areas of health-related practice is the development of ethical frameworks or principles to help guide the actions of practitioners. Familiar examples include the Beauchamp and Childress principles of biomedical ethics ^86^—autonomy, beneficence, non-maleficence and justice—the humanitarian principles ^87^—humanity, impartiality, neutrality and independence—and a variety of public health frameworks which often add to the Beauchamp and Childress principles a number of others including health maximisation, efficiency and proportionality ^88^. The Nuffield Council on Bioethics (NCoB) has proposed an influential ethical framework for public health interventions which states that public health policies should:

- aim to reduce the risks of ill health that people might impose upon each other;
- pay special attention to the health of children and other potentially vulnerable people;
- aim to reduce ill health by regulations that ensure environmental conditions that sustain good health, such as the provision of clean air and water, safe food and decent housing;
- aim to make it easy for people to lead healthy lives by the provision of advice and information.

In doing so, such policies should:

- not attempt to coerce adults to lead healthy lives; and
- seek to minimise interventions that affect important areas of personal life. ^89^

More recently, the NCoB has developed a set of high-level principles—‘an ethical compass’—to govern research into public health emergencies. Such research should be governed by the following widely shared values:

- **Equal respect** – treating others as moral equals, including respecting their dignity, humanity and human rights
- **Helping reduce suffering** – acting in accordance with fundamental duties, founded in solidarity and humanity, to help those in need or suffering from disease; and
- **Fairness** – including both duties of non-discrimination in the treatment of others, and of the equitable distribution of benefits and burdens. ^90^

Although these frameworks, and a range of others like them, identify issues of moral importance in relation to health and climate change, they may not successfully capture all morally-relevant aspects in this field. We have already seen that climate change invites reflection on intergenerational justice and the moral status of the non-human world.

Interestingly, our review identified a small number of articles addressing the need for a principled approach. Singh, in an article mentioned above, listed ten *health* ethics principles to guide decision-making: Stewardship and responsibility; respect for persons; non-maleficence; risk-benefit analysis and burden-identification; reasonableness and relevance; collaboration; least harm; solidarity, duty of rescue, justice and reciprocity; transparency, publicity and engagement, and accountability, appeal and enforcement. These bring together principles to govern substantive, procedural and policy decision-making in relation to health and climate change. Singh also acknowledges that principles drawn from outside the health sphere need to be added, including from the fields of environmental ethics, economic ethics, and climate justice. ^91^ More recently, Gurevich talks of the need for a revised public health framework that gives more acknowledgement to the non-human environment. He posits a ‘restorative commons theory’ that can: ‘bridge environmental ethics and medical ethics by emphasizing the mutual benefits of environmental stewardship to nature and humans. This reflects an expansion of the environmentalist land ethic to a new ‘global health ethic’’. ^92^

In his book on environmental health ethics, Resnik sets out a principle-based method for decision-making, specifically designed to respond to value-conflicts, including those between environmental preservation and economic development, property rights and the public good, and between human and non-human interests. ^93^ In addition to a principle-based method for ethical decision-making involving clarifying the ethical question and resolving conflicts between relevant principles, he proposes the following principles to govern decision-making in this area: ^94^

- Human rights
- Utility
- Justice
- Animal welfare
- Stewardship
- Sustainability
- Precaution.

In 2015, UNESCO (United Nations Educational, Scientific and Cultural Organization) and COMEST (World Commission on the Ethics of Scientific Knowledge and Technology) proposed the following ten principles to regulate responses to adaptation and mitigation in climate change:

- Biological diversity
- Cultural diversity
- Interdependence of life on Earth
- Intellectual and moral solidarity of humankind
- Global justice
- Resilience
- Sustainability
- The precautionary principle
- The duty to share scientific knowledge
- Integrity of scientific research. ^95^

These were later reduced in a 2017 Declaration of Ethical Principles ^96^ to the following six, which form part of an overall statement of broad ethical obligations in response to climate change:

- Prevention of harm
- Precautionary approach
- Equity and justice
- Sustainable development
- Solidarity
- Scientific knowledge and integrity in decision-making.

What all these approaches have in common is an attempt to incorporate normative factors into environmental decision-making, including in environmental health, that stand outside established ethical frameworks in health. Nevertheless, the introduction of factors such as the interdependence of life on earth and the normative claims of the non-human world, present challenges to health frameworks that have been almost exclusively anthropocentric. Work will be required to guide decision-making where human interests collide with moral claims arising from the non-human world. Furthermore, providing guidance for health practitioners and policy makers on how these high-level principles can guide their day-to-day decision-making, particularly when it comes to the compromises demanded of policy, will be critical.

## Data Availability

No data are associated with this article.
