# Supplementary material for: Ethics, climate change and health – a landscape review
Source: Wellcome Open Res. 2023 Aug 14;8:343. [Version 1] doi: 10.12688/wellcomeopenres.19490.1 (PMC10483174; doi:10.12688/wellcomeopenres.19490.1)
Supplement: Peer review history

# Peer review: Reviewer response for version 1

**DOI:** 10.21956/wellcomeopenres.21588.r65248

**Published:** 2023-09-06

**Authors:** John Coggon

**Affiliations:** 1 Centre for Health, Law, and Society, University of Bristol Law School, Bristol, UK

In their paper ‘Ethics, climate change and health – a landscape review’, Julian Sheather and colleagues provide a well-researched and logically-structured analysis of the scale and nature of the challenges for health-oriented ethics research in the context of climate change. These challenges include:

- Appropriately identifying and systematising broad and diverse scientific literatures, and synthesising these against questions of causal and moral responsibility and practical capacity to effect change;
- Establishing appropriate ethical framings for analysis, noting constraints such as basic points of ethical disagreement, the complexities of systems-focused ethical analysis against highly-complex global and historical structures, debates about non-human agents that are rights-and duty-holders (e.g. states, corporations), debates regarding moral concerns for non-human entities in relation to which obligations might arise (e.g. animals, plant life), and considerations overall regarding the limits of theory;
- Achieving principled alignment between real-world practice and otherwise unapplied theory, notwithstanding barriers to embedding ethics in policy that include ignorance/denial of the place of ethics, apparent tensions between political compromise and ethical rigour, and wide-reaching coordination problems.

The paper summarises scientific literatures in climate science, explains the probable effects of climate change, reviews literature on health ethics and climate change, and presents an analysis of a range of ethics frameworks that have been developed to guide practice and policy in this area.

 As the authors explain, even attempting to identify relevant literatures within this wide and fragmented field is a complex task. Given that, the paper offers a very useful approach, which is then well executed and leads to a balanced critique. This effectively outlines the practical challenges, as well as the strengths and deficits in existing literatures. The conclusions are well stated; including on the need not just to develop basic principles, but to provide concrete explanation of how they should guide actors in complex systems in practice.

 Given the necessary—and clearly-explained—methodological limitations, and their inevitable bearing on analysis, I am not of the view that there are problematic claims or omissions in the paper. The nature of the paper is such, however, that it invites identification of further, complementary points that could have been brought into discussion. I raise a small number of these in this review, but recognise that they may be better addressed in future papers rather than the current one.

 Before doing so, I note that Sheather *et al*. explain well various theoretical challenges. These present limitations in health ethics literatures as they exist: in particular, the limited conceptual and analytical applicability of individualistic moral theorising that imagines, essentially, the moral relationship between e.g. a doctor and patient (i.e. ‘standard’ approaches from biomedical ethics). Such approaches do little, insofar as they fail (adequately) to speak to institutional and corporate actors, to populations (including by reference e.g. to citizenship/state residency, socio-economic position, characteristics such as age, disability, gender).

 The authors also do well to explain where health ethics literatures are more apt; notably in the fields of public health ethics and global health ethics. They explain too how these can and should draw from wider areas; in particular environmental ethics. In addressing these points, Sheather *et al*., rightly also note overlaps, as e.g. in Planetary Health and One Health analyses. And they highlight the plurality of academic disciplines that will inform the idea of ‘ethics’ that concerns questions of values, morality, and justice in relation to climate change and health.

 Of interest, one point where a deeper dive could take place is the World Health Organization (WHO)’s own discussions of public health ethics when that field found its modern resurgence; i.e. from around the year 2000. Within those works that aimed to define and provide a basic principled position, the concept of stewardship was central: a point that is notable, amongst other reasons, because of its heritage initially in theology and then in environmental ethics. As is evident, in particular in the final section of Sheather *et al*.’s paper, stewardship still features in literatures, but not with particular (less still universal) prominence. While that may tell its own important story, the WHO’s historical linking of public health ethics and environmental ethics shows a heritage where greater attention could productively be focused. (See e.g. World Health Organization, *World Health Report 2000,* (WHO, 2000); Richard B. Saltman and Odile Ferroussier-Davis, ‘The Concept of Stewardship in Health Policy’, *Bulletin of the World Health Organization* (2000) 78:6, 732-739.)

 A further area where the paper identifies conceptual and theoretical challenges is in health ethics and environmental ethics not being limited to contemporaneous problems and trade-offs. Sheather and colleagues identify considerations in intergenerational justice, both looking backwards in time and forwards. They also neatly summarise some of the greater philosophical challenges that are aired in the literatures; for instance, the so-called ‘non-identity problem’; a challenge whose ‘bite’ I would question in relation to future-facing obligations, especially in the context of population-focused analysis (people may face philosophical objections to complaints about their own existence, but these need not (logically or otherwise) entail a rejection of the legitimacy of complaints that the world around them could and should have been made into a better, fairer place to live). In addition, I would encourage much greater focus in ethical discourses on the idea of ‘health-health tradeoffs’. The authors of the paper correctly point out global tensions e.g. with the wildly unequal spread of causal responsibility, and of who suffers what sorts of harms, where, when, and how. It is important in doing this to emphasise that we are not just interested in how one population’s interests might clash with those of another population, or even about how different costs/harms/benefits can emerge at different points in time. We are also interested in trade-offs between health benefits; i.e. not just (say) health versus negative liberty, or health versus economic gain, but also health versus health. (See e.g. (and in a paper including a focus on environmental regulations) Cass Sunstein, ‘Health-Health Tradeoffs,’ *University of Chicago Law Review* (1996) 63:4, 1533-1571.)

 In short, the paper is pitched around the phenomenon of climate change as a threat to health. But ‘health’ (especially as conceived by the WHO) is not a single thing. This is important, and demands the sort of granular identification of what particular issue is being identified as one of health protection/promotion in a given instance (a point that is not missed by the paper’s authors). In addressing that practically, it is useful to consider formal and strategic synergies and similarities between environmental regulation and (global) public health regulation. In approaching those questions, it is important as well to promote further discussion of the practical nature of obligations that may be identified. Sheather *et al*. do, for instance, mention questions of enforcement. Deep dives are often needed on such questions, framed against realpolitical analysis. Even the existence of a legal obligation does not necessarily mean enforcement is possible (there may be problems of jurisdiction, of standing for potential claimants, etc.) or in practice effective (there may be questions of economic resource, time, and so on). Overall, the review takes a very broad and inclusive understanding of ethics. This is good, but it also means that in analytical and then practical detail specificity is needed about the nature and effect (or lack thereof) of different ‘ethical’ claims and positions, what voices should be heard to gain understanding, and how mediation between equally good/valid, but competing positions can be met.

Is the review written in accessible language?

Yes

Are all factual statements correct and adequately supported by citations?

Yes

Are the conclusions drawn appropriate in the context of the current research literature?

Yes

Is the topic of the review discussed comprehensively in the context of the current literature?

Yes

Reviewer Expertise:

Health, Law, and Society, with particular interests in the philosophy of public health and mental capacity law.

I confirm that I have read this submission and believe that I have an appropriate level of expertise to confirm that it is of an acceptable scientific standard.

# Peer review: Reviewer response for version 1

**DOI:** 10.21956/wellcomeopenres.21588.r65247

**Published:** 2023-08-30

**Authors:** Cheryl Macpherson

**Affiliations:** 1 Department of Clinical Skills, Bioethics Division, St George's University, Saint George's, Saint George, Grenada

Sheather et al are not the first to make the case that policy responses to climate change should be informed by ethical considerations or to highlight ethical frameworks that may strengthen such responses. They and others have pointed out some ethical implications of the health impacts of climate change, the need to navigate and balance conflicting values and priorities in policy decisions, and that bioethics can contribute to such work. The significance of the work by Sheather et al is the high level potential to advance more ethical, health-oriented, and effective policy responses to climate change. The authors draw from recent and reliable evidence to describe the science of climate change, discuss the climate and health literature, identify and discuss key ethical issues regarding climate change, and outline ethical frameworks that may be used in policy responses. Notably, they situate their work as part of a WHO project aiming to embed ethics in climate change policy, practice, and research and thereby produce policy responses that better “address health-related ethical issues”. This is a major step forward for all stakeholders.

 Despite my praise for the work, it has weaknesses. These include the lack distinctions between climate ethics and bioethics and, more importantly, the paucity of references to the literature on climate ethics and bioethics (likewise the sparse references to climate science and health impacts – although this is justified to some extent in the text). This work would be difficult to publish in a bioethics journal because of its reliance almost exclusively on an (excellent) book chapter, monograph, and recent guidance from the Nuffield Council on Bioethics that is not complemented by a deeper discussion of other useful books, journal publications, and resources. To be sure, the ethical references cited put forth seminal ideas and arguments, and define key ethical issues in climate policy responses that continue to warrant attention, but subsequent books and journal publications delve further into these. Even the author most heavily cited (Gardiner) has recent work in a bioethics journal (2022, *Perspectives in Biology and Medicine*).

 This article makes a strong case for the inclusion of ethics in climate and health policy and has potential to catalyze a more interdisciplinary, inclusive, and effective approach to climate policy. That *Wellcome Open Research* is a multidisciplinary resource rather than a bioethics journal, and that the article focuses on policy rather than bioethics, make it acceptable as written. The authors could alleviate concern about the limited references by adding an appendix that cites the ethics references they found (and superficially discussed).

Is the review written in accessible language?

Yes

Are all factual statements correct and adequately supported by citations?

Yes

Are the conclusions drawn appropriate in the context of the current research literature?

Yes

Is the topic of the review discussed comprehensively in the context of the current literature?

Partly

Reviewer Expertise:

Bioethics, climate change, research ethics, public health ethics, low and middle income countries, Caribbean

I confirm that I have read this submission and believe that I have an appropriate level of expertise to confirm that it is of an acceptable scientific standard.

## References

[1] : Environmentalizing Bioethics: Planetary Health in a Perfect Moral Storm. *Perspect Biol Med* .2022;65(4) : 10.1353/pbm.2022.0048 569-585 10.1353/pbm.2022.0048 36468385
